# Knockout of Angiotensin AT_2_ receptors accelerates healing but impairs quality

**DOI:** 10.18632/aging.100868

**Published:** 2015-12-31

**Authors:** Mahya Faghih, Sayed M. Hosseini, Barbara Smith, Amir Mehdi. Ansari, Frank Lay, Ali Karim Ahmed, Tedashi Inagami, Guy P. Marti, John W. Harmon, Jeremy D. Walston, Peter M. Abadir

**Affiliations:** ^1^ Division of Geriatric Medicine and Gerontology, Johns Hopkins University School of Medicine, Baltimore, MD 21224, USA; ^2^ Department of Surgery and Hendrix Burn/Wound Laboratory, Johns Hopkins University School of Medicine, Baltimore, MD 21224, USA; ^3^ Cell Biology Imaging Facility, Johns Hopkins University School of Medicine Baltimore, MD 21224, USA; ^4^ Department of Biochemistry, Vanderbilt University School of Medicine, Nashville, TN 37232, USA

**Keywords:** Angiotensin, AT_1_R, AT_2_R, TGF

## Abstract

Wounds are among the most common, painful, debilitating and costly conditions in older adults. Disruption of the angiotensin type 1 receptors (AT_1_R), has been associated with impaired wound healing, suggesting a critical role for AT_1_R in this repair process. Biological functions of angiotensin type 2 receptors (AT_2_R) are less studied. We investigated effects of genetically disrupting AT_2_R on rate and quality of wound healing. Our results suggest that AT_2_R effects on rate of wound closure depends on the phase of wound healing. We observed delayed healing during early phase of wound healing (inflammation). An accelerated healing rate was seen during later stages (proliferation and remodeling). By day 12, fifty percent of AT_2_R^−/−^ mice had complete wound closure as compared to none in either *C57/BL6* or AT_1_R^−/−^ mice. There was a significant increase in AT_1_R, TGFβ_1_ and TGFβ_2_ expression during the proliferative and remodeling phases in AT_2_R^−/−^ mice. Despite the accelerated closure rate, AT_2_R^−/−^ mice had more fragile healed skin. Our results suggest that in the absence of AT_2_R, wound healing rate is accelerated, but yielded worse skin quality. Elucidating the contribution of both of the angiotensin receptors may help fine tune future intervention aimed at wound repair in older individuals.

## INTRODUCTION

The biology of normal wound healing includes sequential yet overlapping inflammatory, proliferative, and remodeling phases that involve complex biological signaling [1–4]. Dysregulation of specific signaling pathways is thought to underlie skin breakdown and poor healing [1–4]. The renin angiotensin system (RAS) is active in connective tissue and skin, and is known to be important in wound healing [5–7]. RAS is involved in the inflammatory response, collagen deposition and in tissue-related growth factor (TGFβ) signaling necessary for wound healing [5–9]. RAS is known to be dysregulated in both aging and in diabetes, with increased AT_1_R and decreased AT_2_R expression in diabetic wound healing and in aging [5;10], which may play a role in aging skin vulnerability [5;7;8;10]. Indeed, altered dermal AT_1_R and AT_2_R ratio is associated with thinning of epidermis, degeneration of collagen, fracture of dermal layer, and atrophy of subcutaneous fat in diabetic rats [5]. These changes are consistent with those seen clinically in aging skin. AT_1_R blockers impair fibroblast migration and delay wound healing [11]. The angiotensin subtype 2 receptor is less studied, but its anti-inflammatory, anti-apoptotic and anti-proliferative effects are thought to oppose the effects of AT_1_R [12]. Virtually nothing is known about the contribution of the AT_2_R to stages of wound healing. The overarching hypothesis of this study is that a functional balance between skin expression of AT_1_R and AT_2_R is required for optimal healing. We further hypothesized that targeted deletion of AT_2_R would accelerate wound healing rate via un-opposed AT_1_R activity upregulating skin TGFβ signaling. To dissect the role of AT_2_R on wound healing we have selected the genetic knockout to avoid the effects of variations in drug delivery to wound bed. Furthermore, given that Angio-tensin II binds with equal affinity to AT_1_R or AT_2_R, the knockout of the angiotensin receptors allows for better discrimination of the effects of the receptors by eliminat-ing the possibility of remaining unblocked receptors. In this study we compared C57BL/6J wild-type (WT) mice to age- and gender-matched; AT_1_R knockout (*AT_1_R^−/−^*) mice and AT_2_R knockout (*AT_2_R^−/−^*) mice.

## RESULTS

To ascertain the influence of angiotensin receptors on wound healing, downstream effectors and healed skin quality, we compared C57BL/6J wild-type (WT) mice to age- and gender-matched; AT_1_R knockout (*AT_1_R^−/−^*) mice and AT_2_R knockout (*AT_2_R^−/−^*) mice.

### Delayed wound healing in AT_1_R ^−/−^ and accelerated wound healing in AT_2_R^−/−^ mice

**Figure 1:**
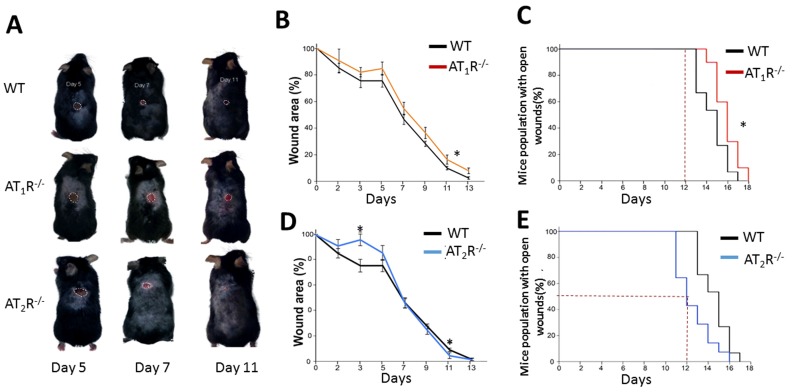
Wound closure measurements in AT_1_R and AT_2_R transgenic mice show delayed AT_1_R^−/−^ and accelerated AT_2_R^−/−^ healing rate. (**A**) Representative images from WT, AT_1_R^−/−^ and AT_2_R^−/−^ mouse cohorts on day 5, 7 and 11 of wound healing. Plannimetric assessment of wound closure rate in AT_1_R^−/−^ (**B**) and AT_2_R^−/−^ (**D**). Complete wound closure of AT_1_R^−/−^ (Panel **C**) and AT_2_R^−/−^ (**E**) mice. Data are means ± SEM *p<0.05.

RAS is a key hormonal system whose dysregulation has been linked to aging, inflammation, and impaired wound healing. We show that the *AT_1_R^−/−^* mice were the most delayed in wound healing as compared to WT and the *AT_2_R^−/−^* (Figure 1, panel A, B and C) which is in agreement with previous reports on wound healing in *AT_1_R^−/−^* mice [11]. In contrast, the healing rate in the *AT_2_R^−/−^ mice was accelerated* which was unexpected given that AT_2_R levels in general correlate with positive outcomes [12]. By day 12, 50% of the animals in the* AT_2_R^−/−^* cohort achieved complete wound healing as compared to none in either the *AT_1_R^−/−^* or WT mice(P<0.05). By day 12, only 5% of the size of wounds remained unhealed in the *AT_2_R^−/−^* mice vs. 17% in the AT_1_R*^−/−^* and 11% in WT mouse cohorts (P<0.05; Figure 1, panel C and E). All the *AT_2_R^−/−^* mice were healed completely by day 16 as compared to 10% of WT and 30% of *AT_1_R^−/−^* that remained with open wounds (P<0.05; Figure 1: panel C and E). Further analysis of the fastest healing group, the *AT_2_R^−/−^*, revealed a delayed healing during the inflammatory phase of wound healing (day1-7) and an accelerated healing during proliferative and remodeling phases (day8-16). (Figure 1D) This biphasic pattern of healing observed in the AT_2_R^−/−^ group, was not seen in either the* AT_1_R^−/−^* or WT mice.

### Increase in wound blood flow in the AT_1_R^−/−^ mice

**Figure 2:**
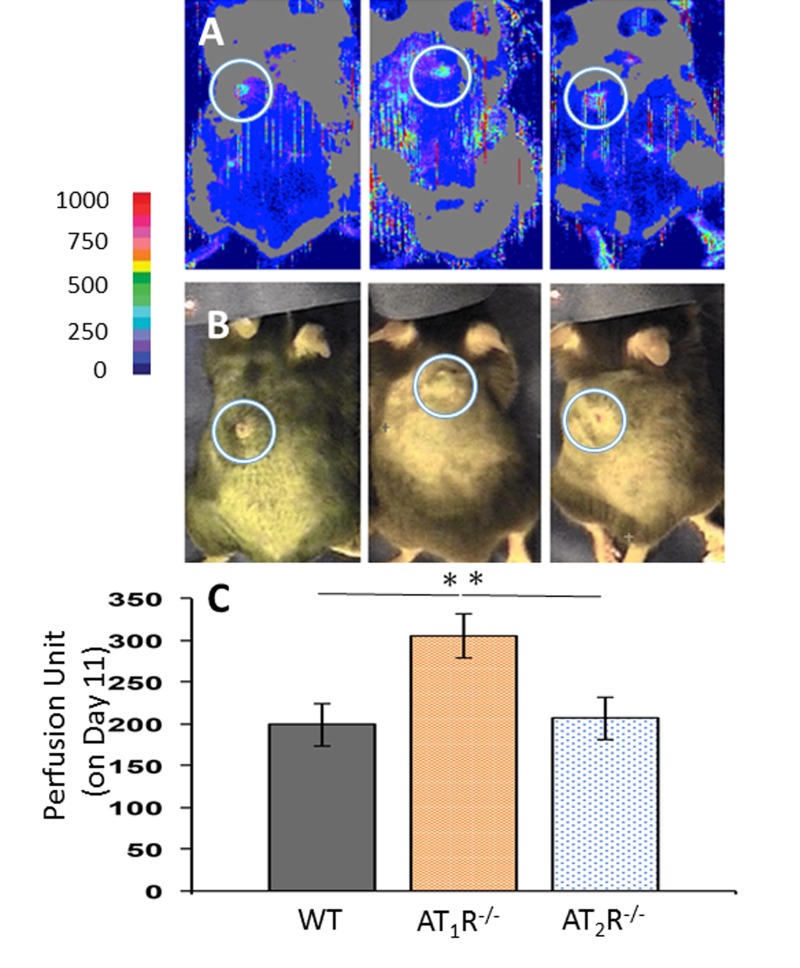
Laser Doppler perfusion imaging of wound area blood flow on day 11 of wound healing shows a higher blood flow in wounds of AT_1_R^−/−^. Data are means ± SEM *p<0.05.

Given the prominent role for angiotensin receptors in tissue perfusion and to determine if changes in blood flow to the wounds contributed to the observed healing pattern in* AT_1_R^−/−^* and *AT_2_R^−/−^* mice, wound area blood flow was measured by non-invasive LDPI on days 7 and 11. There were no differences in wound area blood flow among the three groups by day 7, but by day 11 we observed a significantly higher value in the* AT_1_R^−/−^* as compared to the other two groups *(P<0.05;* Figure 2). Interestingly, the increase in blood flow in the *AT_1_R^−/−^* mice did not correlate with better wound healing. Further, we did not observe differences in wound blood flow the *AT_2_R^−/−^* as compared with WT controls, suggesting that changes in blood flow did not contribute to the accelerated healing observed in the *AT_2_R^−/−^* mice.

### Down regulation of TGFβ isoforms and the downstream target proteins (Smads) *in* AT_1_R^−/−^ and upregulation of AT_1_R, TGFβ_1_ and TGFβ_2_ during later stages of wound healing in the AT_2_R^−/−^

**Figure 3:**
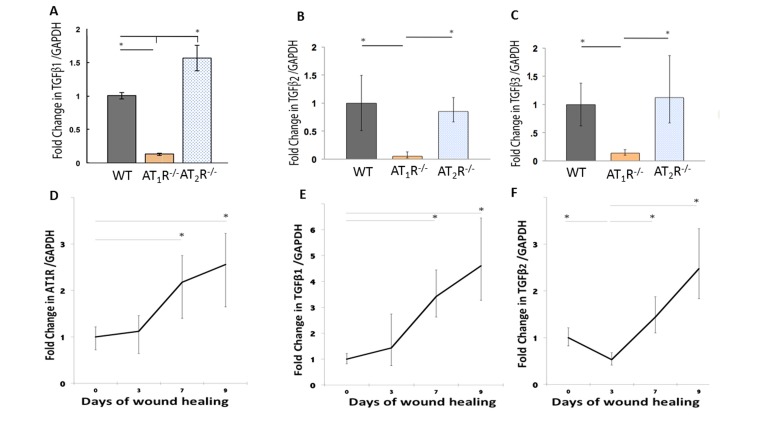
Altered expression of wound TGFβ isoforms in AT_1_R^−/−^ and AT_2_R^−/−^ mice. AT_1_R^−/−^ mice have lower expression of TGFβ_1_ (**A**), TGFβ_2_ (**B**), and TGFβ_3_ (**C**) in healed skin (day20) as compared to WT and AT_2_R^−/−^ mice. An increase in the expression of AT_1_R (**D**), TGFβ_1_ (**E**), TGFβ_2_ (**F**) correlated with the accelerated healing rate observed in later stages of wound healing in AT_2_R^−/−^ mice. The length of fold change error bar equal variance 95% confidence interval *p<0.05.

Although not completely characterized, all phases of wound healing appear to be greatly influenced by subtle modulation of TGF-β, which is strongly influenced by RAS [6;7;30–32]. The three isoforms of TGF-β (β1, β2, and β3) signal through the same cell surface receptor, but appear to play distinct functions during wound healing. While TGF β1 and β2 have predominantly pro-scarring roles, TGF β3 have mainly anti-scarring effects [32]. RAS has been tightly linked to TGFβ activity but the specific effects of AT_1_R and AT_2_R on the different TGF isoforms are not known. Using qPCR we determined differences in expression of the three different isoforms of TGFβ in the healing skin (day 20) of our mouse cohorts. Our results demonstrate a significant decrease in all the three different isoforms of TGFβ in the wound of the *AT_1_R^−/−^ mice* (*P*<0.05; Figure 3: panel A, B and C). TGF-β1 mRNA levels in *AT_1_R^−/−^* mice were decreased 7.69 fold as compared to WT mice. In contrast the TGFβ1 mRNA levels increased 1.83 fold in *AT_2_R^−/−^* mice as compared to WT mice (*P* < 0.005; Figure 3: panel A). The expression of TGFβ2 mRNA was also decreased 20 fold in *AT_1_R^−/−^* mice compared with WT mice (*P* < 0.005; Figure 2: panel B). TGFβ3 mRNA expression was decreased 7.1 fold in *AT_1_R^−/−^* mice in comparison with WT mice (*P* < 0.005; figure 2: panel C). *In contrast, we observed an increase only in TGF*β1 in *AT_2_R^−/−^*, compared to both WT control and *AT_1_R^−/−^* mice (*p* <0.05; Figure 3: panel A). We quantified changes in AT_1_R and the three isoforms of TGFβ in days 0, 3, 7 and 9 of wound healing to investigate if the biphasic pattern observed in wound healing of the *AT_2_R^−/−^* corresponded to a stage dependent changes in AT_1_R and to quantify changes *in* TGF-β isoforms. Our results suggest that the expression of AT_1_R was upregulated by day 7 of wound healing (2.18 and 2.56 fold change respectively, p<0.05; Figure 3: panel D). This increase corresponded to an increase in both TGFβ1 and TGFβ2 (p<0.05; Figure 3: panel E and F). No change in TGFβ3 was observed. Changes in AT_1_R in different stages of wound healing strongly correlated with the changes in TGFβ1 (Pearson r=0.99, p=0.04).

**Figure 4:**
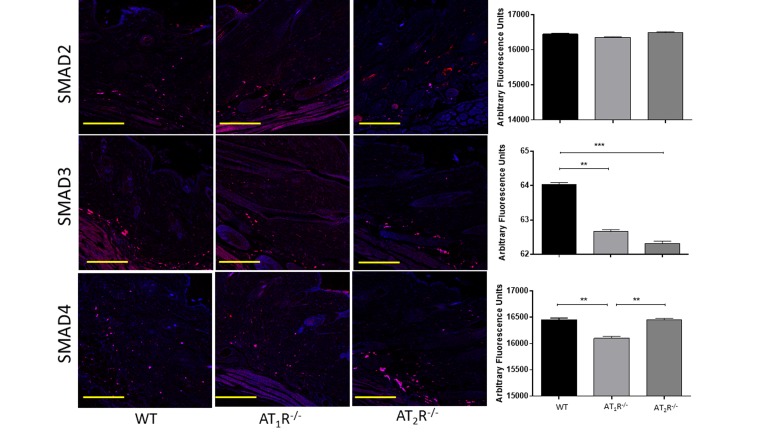
Changes in the TGFβ downstream signaling proteins in wounds of AT_1_R^−/−^ and AT_2_R^−/−^ mice. AT_1_R^−/−^ mice have lower expression of Smad3 and the common mediator Smad4 in healed skin (day20) as compared to WT. A decrease in the expression of Smad3 was also observed in the AT_2_R^−/−^ mice. The photomicrographs presented in red fluorescent staining with a blue DAPI counter stain for nuclei at 10x magnification. Quantification of mean fluorescence intensity of Smads in wild-type and mutant mice is shown. Scale bar 200 μm. **p<0.005, ***p<0.0005.

**Figure 5:**
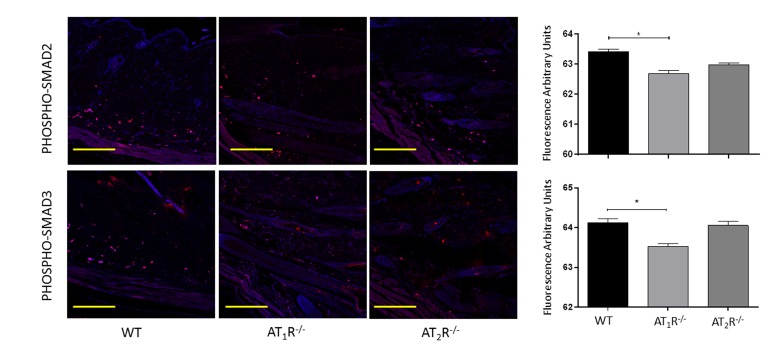
Changes in levels of phosphorylated TGFβ downstream signaling proteins in wounds of AT_1_R^−/−^ and AT_2_R^−/−^ mice. AT_1_R^−/−^ mice have lower expression of Phospho-Smad2 and Phospho-Smad3 in healed skin (day20) as compared to WT. The photomicrographs presented in red fluorescent staining with a blue DAPI counter stain for nuclei at 10x magnification. Quantification of mean fluorescence intensity of phospho-Smads in wild-type and mutant mice is shown. Scale bar 200 μm. *p<0.05.

Next we sought to determine the impact of the disruption of angiotensin receptors on the downstream target proteins of the TGFβ signaling pathway. TGFβ signals through Smad2 and Smad3 that are phosphorylated by TGFβ receptors and translocate to the nucleus with the common-mediator (co-Smad) Smad4 [33;34]. Our results showed no significant difference in Smad2 in wounds of AT_1_R^−/−^ or AT_2_R^−/−^ mice as compared to WT mice (Figure 4). Consistent with the reduction in the three isoforms of TGFβ, we have observed a significant reduction in both Smad3 and the common-mediator Smad4 in wounds of *AT_1_R^−/−^* mice as compared with WT mice (P<0.001, Figure 4). Our results also demonstrate reduction in phosphorylation of Smad2 and Smad3 only in wounds of AT_1_R^−/−^ mice as compared with WT mice (P<0.01, Figure 5). Interestingly, we have observed a similar decrease in Smad3 in AT_2_R^−/−^ mice as compared to WT mice (P<0.0001, Figure 4). There were no differences in Smad4, phospho-Smad2 or phospho-Smad3 in wounds of AT_2_R^−/−^ mice.

### Reduced repair and proliferation activity in wounds of AT_1_R^−/−^ mice

The angiotensin receptors have been linked to changes in cell differentiation and proliferation. While AT_1_R have been shown to increase cell proliferation, AT_2_R have been shown critical for cell differentiation [12].

**Figure 6:**
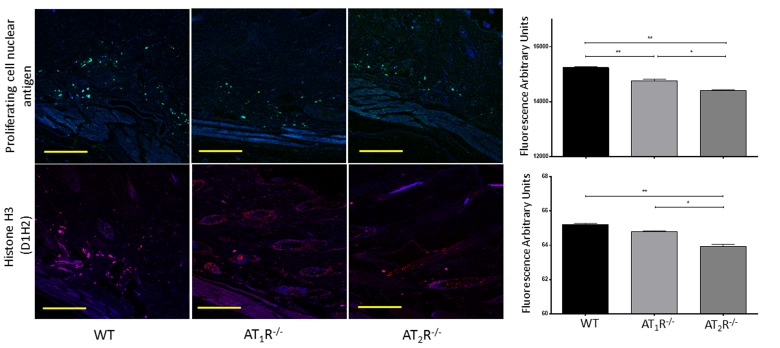
Down regulation of Proliferating Cell Nuclear Antigen in wounds of AT_1_R^−/−^ and AT_2_R^−/−^ mice. AT_2_ R^−/−^ mice have lower expression of total Histone H3 in healed skin (day20) as compared to WT. The photomicrographs presented in green (PCNA) or red (Total Histone H3) fluorescent staining with a blue DAPI counter stain for nuclei at 10x magnification. Quantification of mean fluorescence intensity of PCNA and Histone H3 in wild-type and mutant mice is shown. Scale bar 200 μm. *p<0.05, **p<0.005 .

**Figure 7:**
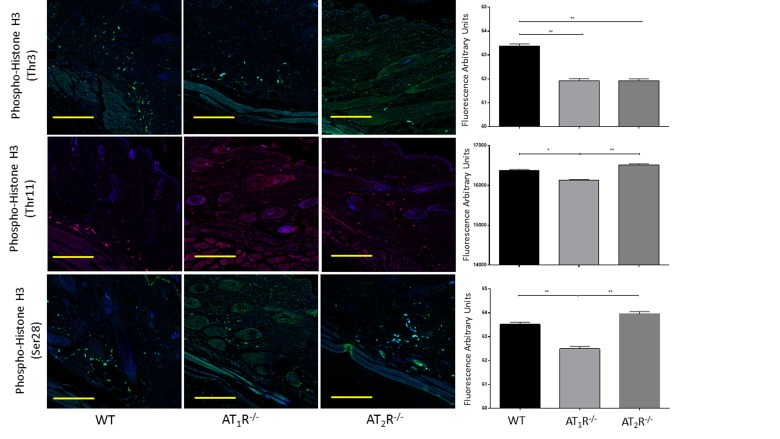
Down regulation of mitotic histone H3 phosphorylation in wounds of AT_1_R^−/−^ at several residues, including serine 28 as well as threonine 3 and 11. AT_2_ R^−/−^ mice have lower expression of Phospho-Histone H3 (Thr3) in healed skin (day20) as compared to WT. The photomicrographs presented in green (Thr3 and S28) or red (Thr11) fluorescent staining with a blue DAPI counter stain for nuclei at 10x magnification. Quantification of mean fluorescence intensity of Phospho-Histone H3 in wild-type and mutant mice is shown. Scale bar 200 μm. *p<0.05, **p<0.005.

Given the observed delayed wound healing noted in AT_1_R^−/−^ mice, we wanted to determine if this delay was driven by changes in cellular proliferative activity. We quantified changes in Proliferating cell nuclear antigen (PCNA), a nuclear protein essential for DNA replication and repair and is a marker for cellular growth and proliferation. To further investigate the mitotic activity at the wound site, we studied expression levels of the phosphorylated form of the histone protein H3. Histone H3 phosphorylation is linked to cells that are actively dividing. Consistent with the delayed healing rate in AT_1_R^−/−^ mice, we have observed a significant reduction in PCNA (P<0.005, Figure 6) and in mitotic histone H3 phosphorylation at several residues, including serines 28 (P<0.005) as well as threonines 3 (0.005) and 11(0.05) (Figure 7). Surprisingly, we have observed a similar decrease in PCNA and phosphorylation of Histone 3 Threonine 3 residue in AT_2_R^−/−^ mice. The impact of the differential phosphorylation of certain histone H3 residues in AT_2_R^−/−^ mice on wound healing activity and scar quality is currently unclear.

### Tensiometry shows wound fragility in AT_2_R^−/−^ mice

Given the pro-scarring and fibrotic effects of TGFβ1, we next sought to determine if there was a difference in the healed skin's physical characteristics (Peak force, total work and compliance). Our results show that despite the accelerated healing rate observed in the *AT_2_R^−/−^ mice*, the healed skin in the *AT_2_R^−/−^ mice* was more fragile, fracturing more easily (Panel 8B), being more compliant (Panel 8C), and breaking with less work (Panel 8D) than wounds from AT_1_R*^−/−^* or WT mice. (P<0.05). Masson's trichrome staining of healing skin shows increase of subcutaneous fat in both AT_1_R*^−/−^* and AT_2_R*^−/−^*. A reduction in dermal collagen zone in AT_1_R*^−/−^* was also observed (Figure 9).

**Figure 8:**
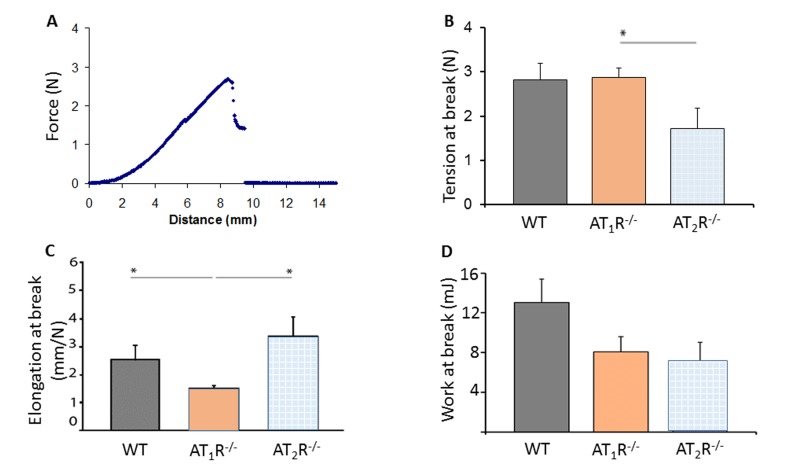
Biomechanical assessment of healed skin in WT, AT_1_R^−/−^ and AT_2_R^−/−^ mouse cohorts. (**A**) Sample representation of tension–elongation curve. (**B**) Comparison of the average tension at the breaking point of mice groups (mean ± SEM, n = 10; *P < 0.05, Mann–Whitney analysis). (**C**) Average elongation at the breaking point of both groups (*P < 0.05, t-test). (**D**) Average work at the breaking point of both groups (calculated from the integral of the curve; *P < 0.05, Mann–Whitney analysis).

**Figure 9:**
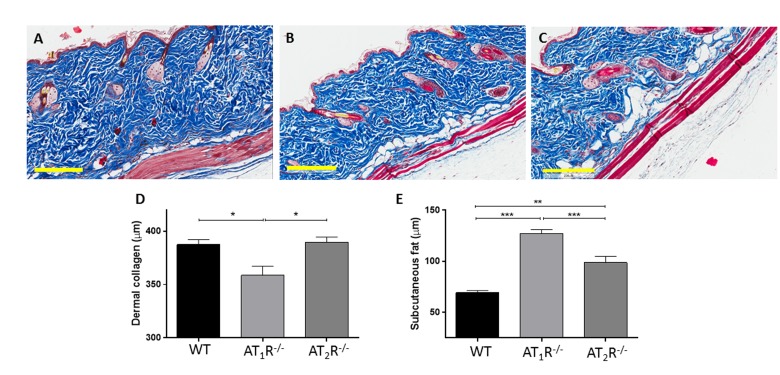
Masson's trichrome staining of skin sections from WT (**A**), AT_1_R^−/−^ (**B**) and AT_2_R^−/−^ (**C**) mouse cohorts shows an expanded zone of subcutaneous fat in the angiotensin knockout mice. Quantification of the thickness of the zones of dermal collagen and subcutaneous fat in wild-type and mutant mice is shown. Scale bar 200 μm. *p<0.05, **p<0.005, ***p<0.0005.

## DISCUSSION

Several lines of evidence suggest that increased RAS activity through the AT_1_R plays a crucial role in wound healing [5–9]. Our results further dissects the impact of angiotensin receptors on wound healing. AT_2_R antagonizes inflammatory signaling, a necessary activating function that leads to the proliferation phase. The lack of AT_2_R was associated with a slower closure rate during the early stages. This may have resulted from an unopposed pro-inflammatory AT_1_R, causing delayed resolution of the inflammatory phase and impairing the transition to the proliferative and remodeling phases [1–4;35].

We have observed a delayed healing pattern in *AT_1_R^−/−^* throughout all phases of wound healing, which is consistent with the pro-inflammatory and pro-proliferative characteristics of *AT_1_R* [12], and is in agreement with previous reports [11]. This is also is supported with the significant reduction in both PCNA and phospho-Histone H3 in healing skin of the *AT_1_R^−/−^* mice. In contrast, by day 8 in *AT_2_R^−/−^* mice, as the healing wounds were transitioning to the proliferative phase we observed a significant upregulation of wound *AT_1_R* along with an accelerated rate of healing. Our combined results of accelerated healing with the upregulation of *AT_1_R* (in *AT_2_R^−/−^* mice), contrasted with delayed healing *in AT_1_R^−/−^* may suggest phase-dependent role for increased *AT_1_R* signaling during the proliferative phase through alterations in TGF-β signaling and alterations in the extracellular matrix [30;36;37].

The relationship between the TGFβ family and angiotensin receptors is not entirely mapped out and remains mechanistically vague. Previous studies have reported that RAS activation through AT_1_R increases TGFβ signaling [38;39]. Our results demonstrate a significant down regulation of all the three isoforms of TGFβ and the downstream targeting proteins Smad3, Smad4, as well as the phospho-Smad2 and phospho-Smad3 in the *AT_1_R^−/−^* mice. The impact of TGFβ on cutaneous wound healing has been well established. The release of TGFβ_1_ during early stages of healing prompts the expression of key components such as fibronectin, collagen types I and III, and VEGF [32]. Additionally, TGFβ_1_ improves angiogenesis to facilitate blood supply to the injured site [40] which then stimulates fibroblasts to allow for wound closure [41]. Whether the decrease in TGFβ_1_ is causal of the impaired wound healing in the *AT_1_R^−/−^* mice is not known. However, in *AT_2_R^−/−^* mice there was a strong, positive correlation between dermal TGFβ_1_ and AT_1_R expression in later stages of wound healing that corresponded to an accelerated wound closure. This is in agreement with previous reports linking AT_1_R stimulation to increased TGFβ_1_ expression and collagen maturation [42] and may potentially explain the decreased compliance seen in healed skin in *AT_1_R^−/−^* mice.

Similarly, the second isoform TGFβ_2_, is involved in granulation tissue formation, angiogenesis and collagen synthesis [43;44]. Impaired wound healing has been demonstrated in TGFβ_2_ transgenic mice [45]. *In AT_2_R^−/−^* mice, we observed a decrease in the expression of TGFβ_2_ by day 3 of wound healing. This initial drop corresponded to the impaired healing seen in early phases in *AT_2_R^−/−^* mice. Furthermore, we have observed a significant increase in TGFβ_2_ by day 7 that was matched with a faster healing rate.

The lack of change in TGFβ_3_ and blood flow in *AT_2_R^−/−^* mice may suggest that these two factors do not play a significant role in modulating wound healing in response to the knocking out of the *AT_2_R*.

Changes in the TGFβdownstream signaling proteins (Smads) have been linked to the rate of wound healing. The down regulation of Smad3 have been linked to acceleration of wound closure [46]. In contrast, the knockdown of Smad4 was associated with aberrant wound healing [47]. Consistent with previous reports on the role of Smad3, we have noted the lowest level of Smad3 in *AT_2_R^−/−^* along with the fastest wound closure rate. The down regulation of Smad4 in *AT_1_R^−/−^* may have played a role in the delayed healing rate.

In summary, the silencing of AT_1_R delayed wound healing, while the interruption of AT_2_R accelerated wound healing. Furthermore, this effect in *AT_2_R^−/−^* mice, was at least partially mediated by *AT_1_R*, TGFβ_1_ *and* TGFβ_2._ This data supports the notion of the antagonistic interaction between AT_1_R and AT_2_R.

Mitochondria provide energy and produce reactive oxygen species to drive the increased mitotic and synthetic activity necessary for wound healing. Several groups demonstrated a link between age-related mitochondrial dysfunction and impaired wound healing [48]. The identification of a functional intra-mitochondrial angiotensin system (MAS) [49] may provide additional insight into the RAS interface with wound healing. Activation of the intra-mitochondrial AT_2_R is coupled to increased nitric oxide generation and inhibition of mitochondrial energy production [49]. Further work is needed to determine the impact of MAS on wound healing.

There are several limitations to our current study. Structurally, mice skin differs from human skin in that mice have much thinner epidermal and dermal layers than humans. Furthermore, mice also have a large subcutaneous muscle layer, which augments wound repair by contraction making further studies in a second animal model (pigs) or humans necessary before extrapolating results to humans. Also, given that we are studying mice that are homozygote knockouts for either the AT_1_R or the AT_2_R, partial effects of the genes and the compensatory effects of one angiotensin receptor on the absence of the other receptor are still not clear.

Given the effects of aging on angiotensin receptors [12;49;50], and that many aged, frail individuals are already on angiotensin receptor blockers, this research highlights the crosstalk between AT_1_R and AT_2_R and that pinpointing the exact molecular changes in angiotensin receptors and the impact of angiotensin receptor blockers on wound healing in aged individuals is important for the progression of the field of wound healing.

## METHODS

### Mouse models

This study was approved by the Johns Hopkins Animal Care and Use Committee (ACUC). To ascertain the influence of angiotensin receptors on wound healing, downstream effectors and healed skin quality, we compared 28-week old male C57BL/6J wild type (WT) mice (Jackson Laboratories, Bar Harbor, Maine) to age and gender matched AT_1_R knockout (AT_1_R^−/−^) (Jackson Laboratories, Bar Harbor, Maine) [13] and AT_2_R knockout (*AT_2_R ^−/−^* mice (supplied by our collaborator Dr. Tedashi Inagami, Vanderbilt University, TN) [14;15]. Male mice were employed to avoid the effects of hormonal changes on wound blood flow and healing.

### Wounding procedure and area calculation

Mice (N=10 in each group) were anesthetized by a mobile RC^2^ non-rebreathing anesthesia machine (Vet Equip, Inc. Pleasanton, CA). Buprenorphine (1 mg/kg) was administered by a subcutaneous injection during the first 24 hours. A full thickness 8 mm wound was created by punch biopsy. On days 0, 3, 5, 7, 9, 11 and 13, the wound borders were traced *in situ* onto clear acetate paper. Images were digitized at 600 dpi (Hewlett Packard Company, Laser Jet 3390, Paolo Alto). Wound areas (in pixels) were calculated using Adobe Photoshop CS3 Image software (Adobe System Inc. San Jose, CA). Wound area on day 0 was taken as a 100% and a wound size ratio obtained with that measurement in each time point. The wounds were checked daily after day 10 until complete closure.

### Laser Doppler Perfusion Imaging (LDPI)

Blood flow in the wound areas was measured at days 7 and 11 using a 633 nm, He–Ne scanning laser Doppler imaging device (Moor Instruments, Devon, UK], which utilizes a near-infrared laser diode to measure subcutaneous blood flow as a function of light scattering by moving red blood cells (Doppler shift), as described previously [16].

### Physical measurements of tissue strength

Peak force, work to rupture and flexibility of healed skin were calculated at day 21 using a FGV-10XY tensiometer (Checkline by Electromatic, Cedarhurst, NY) to record the force generated as the skin was elongated until rupture as described previously [17].

### Histology/Immunofluorescence

Healing skin tissues were embedded in Tissue-Tek O.C.T. Compound (Sakura) and multiple thin sections (5 μm) were cut using a cryostat (Microm). Subsequently, the sections were stained with Masson's Trichrome (Polysciences, Inc.) or using immunofluorescence techniques. Masson's Trichrome staining was carried out according to the manufacturer protocol with the addition of an one hour 10% formalin fix at room temperature (RT) prior to the fixation in Bouin's solution (Meinen 2011). Slides were then digitally scanned and under high-power fields both dermal and subcutaneous fat thicknesses were measured [18] using Aperio ImageScope software (Leica Biosystems, Germany). For immunostaining, the sections were fixed with 4% paraformaldehyde for 15 minutes at RT then blocked with 5% BSA/0.3% TritonX-100/PBS for one hour at RT, incubated with the primary antibodies, overnight at 4°C. For the TGFβsignaling cascade, the following antibodies were purchased commercially: anti- Smad2 (D43B4) rabbit mAb (1:100 dilution, Cat#5339) [19], anti- Smad2/3 (D7G7) Rabbit mAb (1:100 dilution, Cat# 8685) [20], anti- Smad3 (C67H9) rabbit mAb (1:100 dilution, Cat# 9523) [21], anti- Smad4 (cat#9515) [22], anti-Phospho-Smad2 (Ser465/467) (138D4) rabbit mAb (1:100 dilution, cat#3108) [23], and anti-phospho-Smad3 (Ser423/425) (C25A9) rabbit mAb (1:100 dilution, Cat#9520) [24] from Cell Signaling Technology, Beverly, MA. For cell proliferation activity, the following antibodies were purchased commercially: anti- Proliferating cell nuclear antigen (PCNA) PCNA (1:2400 dilution, cat# #8607) [25], anti- Histone H3 (D1H2) rabbit mAb (1:100 dilution, Cat#4499) [26], anti-phospho-Histone H3 (Thr3) (cat#9714) [27]; anti- phospho-Histone H3 (Thr11) (cat#9764) [28], anti- phospho-Histone H3 (Ser28) (1:100 dilution, cat# 9713) [29] from Cell Signaling Technology, Beverly, MA. Sections were then incubated with secondary IgG (H+L), F(ab')2 Fragment (Alexa Fluor® 555 Conjugate, (1:1000 dilution, Cat #44091) at RT for 1 hour. Slides were mounted with Vectashield Hard Set with Dapi (Vector Laboratories). All images were taken at 10x, 0.3 NA, PlanNeoflaur on the Zeiss LSM 700. Imaging and quantification were done by the JHU microscope facility by blinded examiners. Automated quantification of mean intensity of fluorescent signal were done using Volocity image analysis software (v6.3, Perkin Elmer, Waltham, Massachusetts).

### Quantitative real-time reverse transcription PCR

Total RNA was extracted from half of each wound sample using TRIzol (Invitrogen, Frederick, MD, USA), RNeasy Kit (QIAGEN, Redwood City, CA, USA), based on the manufacturer's protocol. Single-stranded cDNA is synthesized using iScript cDNA Synthesis Kit (Bio-Rad, Hercules, CA, USA). The fold changes in TGF-β 1 (primer sequence: Fwd: 5′GAGCCCGAAGCGGACTACTA 3′; Rev: 5′ CCCGAATGTCTGACGTATTGAAG 3′), TGF-β 2 (primer sequence: Fwd: 5′ AGAATCGTCCGCTTTGATGTC 3′; Rev: 5′ TCTGGTTTTCACAACCTTGCT 3′), TGF-β 3 (primer sequence: Fwd: 5′ CAGGCCAGGGTAGTCAGAG 3′; Rev: 5′ ATTTCCAGCCTAGATCCTGCC 3′.) gene expression of healed tissue samples was normalized to Glyceraldehyde-3-phosphate dehydrogenase (GAPDH) using the threshold cycle for amplification as $2^{-\Delta \Delta C_{T}}$, where as $\Delta \Delta C_{T}=\Delta C_{T,Control}-\Delta \;C_{T,T\arg et}$. Real-time PCR was performed using Brilliant II SYBR Green QPCR Master Mix (Agilent Technologies, Santa Clara, CA, USA) and Agilent Mx3000P QPCR Systems (Agilent Technologies, Santa Clara, CA, USA).

### Statistical analysis

Data were presented as mean ± SEM. Differences in means between groups were analyzed for significance using two-way analysis of variance, followed by Holm-Sidak post hoc analysis when appropriate. Complete closure rate as a final outcome, was assessed with the Kaplan-Meier method, using Gehan-Breslow test to determine differences. Student's t-test and Mann–Whitney test were also used to analyze the biomechanical tensiometry data. A probability value of <0.05 was considered statistically significant.

## Abbreviations

Ang II: angiotensin II
AT_1_R: Angiotensin II type 1 receptors
AT_2_R: Angiotensin II type 2 receptors
AT_1_R^−/−^: Angiotensin II type 1 receptors knockout
AT_2_R^−/−^: Angiotensin II type 2 receptors knockout
TGFβ: transforming growth factor-β
